# XSTREAM: A practical algorithm for identification and architecture modeling of tandem repeats in protein sequences

**DOI:** 10.1186/1471-2105-8-382

**Published:** 2007-10-11

**Authors:** Aaron M Newman, James B Cooper

**Affiliations:** 1 Biomolecular Science and Engineering Program, University of California, Santa Barbara, CA 93106, USA; 2 Department of Molecular, Cellular, and Developmental Biology, University of California, Santa Barbara, CA 93106, USA

## Abstract

**Background:**

Biological sequence repeats arranged in tandem patterns are widespread in DNA and proteins. While many software tools have been designed to detect DNA tandem repeats (TRs), useful algorithms for identifying protein TRs with varied levels of degeneracy are still needed.

**Results:**

To address limitations of current repeat identification methods, and to provide an efficient and flexible algorithm for the detection and analysis of TRs in protein sequences, we designed and implemented a new computational method called XSTREAM. Running time tests confirm the practicality of XSTREAM for analyses of multi-genome datasets. Each of the key capabilities of XSTREAM (e.g., merging, nesting, long-period detection, and TR architecture modeling) are demonstrated using anecdotal examples, and the utility of XSTREAM for identifying TR proteins was validated using data from a recently published paper.

**Conclusion:**

We show that XSTREAM is a practical and valuable tool for TR detection in protein and nucleotide sequences at the multi-genome scale, and an effective tool for modeling TR domains with diverse architectures and varied levels of degeneracy. Because of these useful features, XSTREAM has significant potential for the discovery of naturally-evolved modular proteins with applications for engineering novel biostructural and biomimetic materials, and identifying new vaccine and diagnostic targets.

## Background

Repeated sequences, often organized as extended tandem arrays, abound in biology, and computational approaches have been critical for the identification and analysis of such sequence elements from genomic data. Tandem Repeats (TRs) are formally defined as two identical copies of finite non-empty words with no intervening characters [1]. Since biological sequences evolve naturally by mutation, both by base substitutions and insertions/deletions (indels), a biological TR is defined as two or more *sufficiently similar *biological words lacking intervening characters, where sufficiency is arbitrarily defined. The work described in this paper focuses exclusively on non-evolutionary TRs (for evolutionary TR detection, see [2]), each of which has three important properties: *consensus sequence*, a word representing the TR pattern, *period*, the number of characters in the consensus sequence, and *copy number*, the number of words in the entire TR domain.

Bioinformatics studies of TRs have primarily focused on DNA. DNA TRs are traditionally classified on the basis of increasing period into microsatellites, minisatellites, and large-scale duplications. In some human TR loci, copy number changes are associated with triplet-repeat expansion diseases that include Huntington's disease and Fragile X Syndrome [3]. Because genomic TR loci are often highly polymorphic, even expanding and contracting from generation to generation, DNA TRs have forensic and biomedical applications, and may play important roles in genome evolution [4,5].

Nucleotide repeats occurring in protein coding genes can result in protein sequences containing repetitive elements. Though less studied than DNA repeats, peptide repeats are likewise known to be widespread in nature [6-8]. Peptide TRs impart a modular architecture to proteins and are found in important structural proteins such as animal collagens and keratins, insect and spider silks, plant cell wall extensins, and the proteins that form adhesive plaques and byssal threads of bivalve mussels [9-13]. TR domains are also found in other modular proteins, including prion proteins, ice nucleation and antifreeze proteins, FG-rich proteins in nuclear pore complexes, surface antigens of microbial pathogens and parasites, histones, and zinc-finger transcription factors. [14-20]. Peptide TRs may provide an evolutionary shortcut for the modular construction of new proteins through recombination and copy number adjustment [6,7,21,22]. To understand both the evolutionary diversity and functional significance of protein TRs, facile methods for the *a priori *identification and analysis of TRs from protein sequence databases will be critical.

Numerous bioinformatics tools have been developed for *de novo *repeat detection in DNA and protein sequences. One class of tools utilizes sequence self-alignment (SSA) [23-26]. Importantly, SSA approaches allow for the substitutions and indels in repeat sequences that often arise in biology. Because protein repeat detection tools that use SSA (RADAR, TRUST, Pellegrini et al. method) detect all repeated sequences, not only TRs, these algorithms may incorrectly characterize TR domains as non-TRs. With Ω(*n*^2^) time complexity (where *n *= length of input sequence), SSA algorithms are less than ideal for long protein sequences and repeat-detection in large multi-genome datasets. An alternative strategy implemented for *a priori *peptide repeats detection is based on a sliding window (SW) approach [22,26-28]. In general, SW algorithms are simple to implement, but do not readily accommodate indels and are thus likely to miss many degenerate TRs. The Ω(*n*^3^) time complexity of SW algorithms used to detect repeats of all periods also renders this strategy inappropriate for analysis of long sequences.

An efficient heuristic employed for detecting DNA TRs in whole genome data relies on seed extension (SE) [29,30]. Seed extension algorithms have Ω(*n*) time complexity for repeat detection, and depending on implementation, can approximate O(*n*) time complexity, making them fast enough for analyses of large sequence databases. Furthermore, since SE allows for both indels and substitutions, this method is very appropriate for repeat finding applications in naturally evolving biological sequences.

To complement and improve upon current software tools for peptide repeat detection, we implemented a SE algorithm to explicitly locate exact and degenerate (with substitutions and indels) TRs of all periods in protein sequences. This new tool, called XSTREAM for Variable ('X') Sequence Tandem Repeats Extraction and Architecture Modeling, was designed to efficiently mine large genomic datasets for TRs of any period, to effectively characterize degenerate TR domains, and to produce concise TR output. Important features of XSTREAM include novel heuristics that achieve 1) practical running time without period limitations, 2) effective reduction of TR output redundancy, 3) merging of discontinuous degenerate TR domains, 4) identification of nested TR architectures, and 5) TR domain clustering. Though developed specifically for analyzing TR protein sequences, XSTREAM works equally well to extract TR patterns in DNA sequences, or for that matter, TRs in any ASCII string of characters. The practical utility of XSTREAM is demonstrated through testing and validation using publicly available genome sequence data.

## Implementation

The XSTREAM program implements a SE approach that includes heuristics to efficiently and effectively detect exact and degenerate TRs of any period from large input sequence datasets. The program utilizes two important strategies in addition to SE to achieve practical running times without period limitations: a user-modifiable sequence alignment method called Gap-Restricted Dynamic Programming (GRDP), and a new long-period TR filter (both described in the Appendix). In addition, XSTREAM applies several strategies, including the use of *irreducible *repeats, to effectively combat the redundancy in TR detection inherent in biological TR sequences. Other novel features incorporated into XSTREAM include merging of degenerate TR domains and modeling of nested TR architectures. XSTREAM provides non-redundant output of TRs meeting a suite of user-defined criteria for attributes such as minimum and maximum period, minimum copy number, minimum domain length, minimum % input sequence coverage, and maximum character mismatch.

### Algorithm

**Figure 1:**
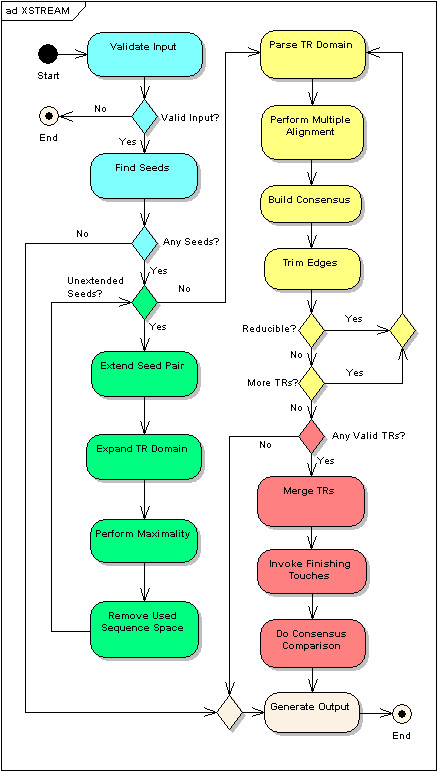
**XSTREAM Program Flow Chart**. Activity Diagram of XSTREAM modeled using Enterprise Architect version 4.10.739 (Sparx Systems).

The primary functionalities of XSTREAM, as shown in Figure 1, can be divided into five high level stages: Pre-Processing, TR Detection, TR Characterization, Post-Processing, and Output. For a technical description of the algorithm, presented within the same organizational context, refer to the Appendix section.

### Pre-Processing

For processing by XSTREAM, input sequences must be in FASTA format. Valid sequences are sent to the seed detection module. XSTREAM searches the input sequence for short exact substring repeats, or seeds, of two or three sizes, depending on the input length (see [29] for an excellent example of the use of seeds, or *k*-tuple *probes *in TR detection). Seed pairs are used to provide starting points and potential periods for TR detection. The use of seeds allows XSTREAM to rapidly identify putative TRs. For every adjacent pair of matching seeds, XSTREAM records both the sequence distance between them and the sequence index of the leftmost seed. Each distance is a potential TR period.

### TR Detection

Following seed detection, XSTREAM attempts to extend each seed pair. Two sequence iterators move downstream from each seed in a parallel manner, returning characters for comparison. Running totals of character match and mismatch are kept. We define *i *as the amount of character matching required between two tandemly arranged words in order for them to be designated a TR. For example, if *i *is set to 0.8, then at least 80% of the aligned characters among two words at a given period must be identical. Seed extension always stops when for any seed pair, the iterator for the leftmost seed collides with the rightmost seed. If at any point during the procedure, the character mismatch count divided by the current potential period exceeds or equals 1 - *i*, seed extension is aborted, thereby reducing running time. Similarly, seed extension is prematurely terminated if the match count becomes sufficiently high. To include indels during seed extension, we use a novel heuristic, which is presented in the Appendix section.

Each candidate TR resulting from successful seed extension is subjected to further expansion using the same basic mechanism as seed extension. XSTREAM examines sequence space both downstream and upstream of the current candidate domain using increments equal to the TR period. Potential repeat copies are evaluated by comparing new sequence space with the reference repeat, which is the leftmost repeat resulting from the initial seed extension. If indels are allowed and if domain expansion using seed extension fails to agree with *i*, we invoke a second strategy. The second approach, termed GRDP (see Appendix), can more accurately perform a subsequence pairwise comparison at the expense of slightly increased running time. A novel feature of our implementation is the user's ability to limit the maximum width of the dynamic programming (DP) matrix (parameter *g*), resulting in θ(*n*) time and space complexities for global pairwise alignments.

Following domain expansion, we instantiate a procedure called maximality. Employing a user-adjustable scoring scheme, maximality finds the longest stretch of characters both downstream and upstream that can legitimately be added to each candidate TR. This procedure is invoked because TRs in nature do not always occur in integer copy numbers and XSTREAM's TR domain expansion method is limited to integer copies.

Finally, XSTREAM masks input sequence space corresponding to each maximally extended candidate TR. Sequence masking prevents further seed extensions in sequence regions that constitute TR domains, thus functioning to prevent output redundancy as well as reduce running time. For details of sequence masking, refer to Redundancy Elimination I as well as Two-stage TR detection in the Appendix.

### TR Characterization

To further refine each candidate TR, XSTREAM segments every TR domain into its component copies. Parsing can be accomplished by a trivial subdivision of the TR domain using the current period, an optimal subdivision using wrap-around dynamic programming (WDP, [31]), or a heuristic subdivision using GRDP. For details about implementation and when each method is invoked, refer to the Appendix section.

Following TR parsing, each TR undergoes a multiple alignment of its copies. A procedure identical in concept to STAR Alignment is used when indels are allowed. Because practical running time is emphasized in our implementation, pairwise sequence comparisons during STAR Alignment may be computed in a non-optimal manner using GRDP.

Following multiple alignment of each TR, a consensus sequence is computed. Each consensus is democratically derived using the majority rule. In addition, XSTREAM computes an error associated with the consensus – the lower the error, the stronger the agreement between the consensus and its represented domain. We define *I *as the minimum allowable matching between the consensus and the aligned TR for the TR to be reported to the user. For example, if *I *equals 0.8, then the consensus error cannot exceed 0.2 or 20% disagreement.

Next, XSTREAM inspects the edges of each aligned TR domain (with TR copy number greater than 2) for accordance with the consensus. If either edge mismatches with the consensus, that edge is truncated. Since all TRs must have at least 2 copies, edge trimming is not performed on TR domains with TR copy number = 2.

Occasionally, because of matching considerations, TR domains are identified with periods that are reducible. Therefore, the last step of TR Characterization functions to reduce overestimated TR periods (see Redundancy Elimination II in the Appendix).

### Post-Processing

XSTREAM attempts to merge *sufficiently similar *TRs that either overlap in the input sequence or are in close enough proximity to one another. To compute sufficient similarity, XSTREAM invokes the concept of cyclical permutations, which enables effective consensus sequence comparison (see *Merging *and *Consensus Comparison *in the Appendix). As a result, XSTREAM can identify TR domains with large regions of indels and/or substitutions that, without merging, would be reported as separate TRs. This procedure is thus important for detecting rapidly evolving TR sequences.

Following merging, XSTREAM invokes a series of finalizing functions called finishing touches, which serve to fine-tune the characterization of each TR domain as well as remove TRs that are insufficiently fit for output. TR characterization refinement involves rerunning maximality, redoing multiple alignment, rerunning reducibility, and looking for nested TRs (see Appendix). After additional characterization, finishing touches removes TRs with unacceptable amounts of overlap (see Redundancy Elimination III in the Appendix). Finally, remaining TRs are tested for agreement with user-defined filtration criteria.

All TRs that satisfy the output criteria are sent to the consensus comparison (CC) module. CC clusters TRs on the basis of consensus similarity. By ordering TRs by consensus sequence homology in the output, XSTREAM reduces output redundancy while facilitating the identification of TR families from the input dataset. Related TRs may reflect structural or functional homology of their corresponding protein sequences. The current implementation of CC only compares TRs of equal period.

### Output

XSTREAM automatically generates HTML files in a format similar to the output from Tandem Repeats Finder (TRF) [29]. HTML output 1 contains a TR summary table and list of TR information, including sequence positions, period, and copy number. The range of sequence positions for each TR is hyperlinked to HTML output 2, which displays TR multiple alignments and consensus sequences. In the case of a multiple sequence input, XSTREAM generates HTML output 3, which reports a list of all input sequences containing reported TRs. An additional output option is a colored TR schematic, in PNG or HTML format, that represents the modular architectures of TR-containing sequences. The main user-definable output parameters of XSTREAM are presented in Table 1. A list of all user-defined parameters can be found on the XSTREAM webserver [32].

**Table 1:** User-defined parameters

**Definition**	**Default Value**
Minimum character identity *i*	0.7 for proteins 0.8 for nucleotides
Minimum consensus matching *I*	0.8
Minimum copy number *MinC*	3
Minimum period *MinP*	3 for proteins 10 for nucleotides
Maximum period *MaxP*	Half of input sequence length
Maximum consecutive gaps *g *(see Appendix)	3
Maximum indel error (see Appendix)	0.5

Shown in this table are seven important user-adjustable parameters used by XSTREAM. These parameters function to limit the extent of TR degeneracy as well as to restrict the TR period and copy number of reported TRs. Default parameter values were empirically chosen to preferentially identify and model long degenerate repeat regions rather than shorter repetitive regions with higher sequence identity (e.g., where *I *= 1.0 and *g *= 0). We acknowledge that alternative architectures may exist for some complex repetitive domains. By including these and additional modifiable parameters, XSTREAM provides considerable user control over TR degeneracy and output filtration.

## Results

XSTREAM was coded using Java Standard Edition 5.0. To evaluate our implementation, we demonstrated and validated key features of XSTREAM using a variety of input datasets. First, a run time analysis shows the practicality of XSTREAM for TR detection in whole genomic sequence data. Second, multiple sequence alignments, merging, and nesting are demonstrated using anecdotal output examples. Third, the ability of XSTREAM to detect protein TR domains is validated using published results from five protozoan parasite genomes. Finally, we present schematic diagrams illustrating the utility of XSTREAM for graphically depicting modular architectures of TR proteins. In all cases, default parameter values were used unless stated otherwise (see Table 1). All tests and data collection were carried out using a Windows XP PC with a 64-bit AMD Athlon dual core 1.8 Ghz processor and 2 Gb RAM.

A principle attribute of XSTREAM is practical running time for large sequence datasets. To measure how running time varies with differing input sequence lengths and parameter values, we used XSTREAM to analyze DNA sequences. We chose DNA over protein sequences simply because DNA sequences cover a substantially larger range of sequence lengths than proteins, thus enabling a more accurate assessment of running time. XSTREAM was run on DNA sequences ranging from 0.23 Mbp to 202 Mbp, either with gaps (*g *= 3) or without gaps (*g *= 0). For these analyses, sequences were examined in two sets. Shorter sequences, < 10 Mbp, were processed with minimum TR domain length *minD *= 20 and minimum period *MinP *= 1, and no period restrictions. For longer sequences, we used *minD *= 50 and *MinP *= 10, and due to memory limitations, maximum period was set to 100 kbp. In addition, for periods 10 – 999 we used a divide-and-conquer approach (see Appendix) with fragment length = 1 Mbp. As shown in Table 2, running time increased approximately linearly with increasing sequence length for all DNA sequences with or without gaps (R^2 ^> 0.99). Next, the effect of increasing dataset size on running time was examined by analyzing four Swiss-Prot datasets ranging in size from 40,292 to 230,150 non-redundant protein sequences, and setting *minD *= 10 and *MinP *= 1. As expected, since XSTREAM processes each protein sequence individually, running time scaled linearly (R^2 ^> 0.998), as indicated in Table 2. A running time of less than 7. 5 min for the detection of degenerate TRs (using *g *= 3) from the Swiss-Prot 50.5 dataset clearly demonstrates the practicality of XSTREAM for multi-genome data mining.

**Table 2:** Running Time Analysis

**Source**	**Length, Mbp**	**Time, min *g *= 3**	**Time, min *g *= 0**	**Longest period**
*S. cerevisiae *Chr. I	0.23	0.25	0.12	135 (17.9)
*S. cerevisiae *Chr. VIII	0.56	0.58	0.29	1998 (2)
*H. sapiens *β TCR	0.68	0.77	0.36	340 (2)
*S. cerevisiae *Chr. XII	1.0	1.2	0.49	9137 (2)
*M. magneticum *AMB-1	4.9	6.4	2.2	1158 (4.2)
*H. sapiens *Chr. I contig	9.8	13.5	4.7	18557 (2.1)

**Source**	**Length, Mbp**	**Time, min *g *= 3**	**Time, min *g *= 0**	**Longest period**

*H. sapiens *Chr. XXI	33.0	34.4	16.4	3379 (2)
*R. norvegicus*	80.7	86.7	39.1	2715 (2)
*H. sapiens *Chr. X	127.6	134.7	64.1	4863 (2)
*M. musculus *Chr I	202.5	239.1	90.0	3773 (2)

**Source**	**No. of Proteins**	**Time, min *g *= 3**	**Time, min *g *= 0**	**# TRs (# TRPs)**

Swiss-Prot v.30	40292	1.5	0.55	2428 (3771)
Swiss-Prot v.38	80000	2.6	1.1	3762 (7012)
Swiss-Prot v.45	163633	5.4	2.4	5302 (12359)
Swiss-Prot v.50.5	230150	7.3	3.5	6444 (17097)

Running times for the analysis of different input sequence datasets are shown, with the gap parameter *g *= 3, or *g *= 0. The following DNA sequences were downloaded from NCBI: *S. cerevisiae *Chromosomes I (gi 85666109), VIII (gi 82795252), and XII (gi 85666119), *H. sapiens *Chromosomes X (gi 89033689) and XXI (89058287), Chromosome I contig (gi 29789880), and the β T-cell receptor locus (gi 114841177), *R. norvegicus *Chromosome XVI (gi 109504251), *M. musculus *Chromosome I (gi 83274080), and the *M. magneticum *AMB-1 (gi 82943940) genome. Sequences at the top (0.23 – 9.8 Mbp) were run with *minD *= 20, minP = 1, and all possible maximum periods. Longer DNA sequences (33 – 202.5 Mbp) were run with *minD *= 50, *minP *= 10, and (due to memory limitations) maximum period = 100 kbp; divide-and-conquer (see Appendix) was used for periods < 1000 (fragment length = 1 Mbp). For each longest period found, the copy number is shown in parentheses. These data show a linear relationship between running time and increasing input sequence length (R^2 ^> 0.99). Running times for analysis of 4 Swiss-Prot datasets, using *minD *= 10 and *minP *= 1, shown at the bottom, including the number of TRs detected (using consensus comparison, see Appendix) and the number of TR-containing proteins found (in parentheses). XSTREAM running time scaled linearly with increasing Swiss-Prot dataset size (R^2 ^> 0.998).

**Figure 2:**
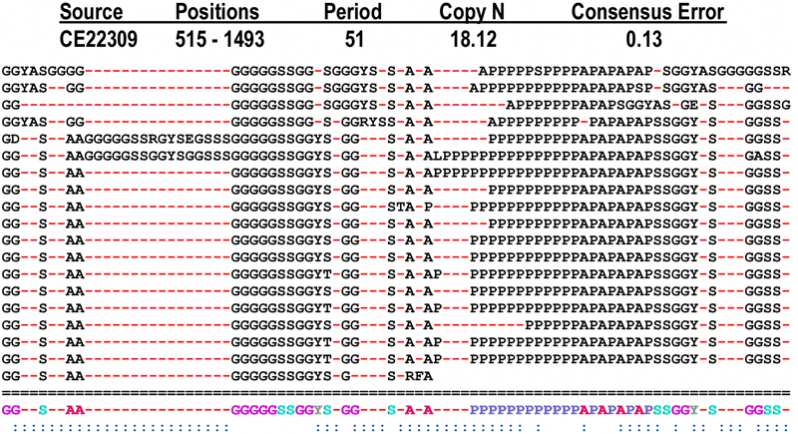
**Multiple Alignment of TR domain from *C. elegans***. Standard TR properties are shown above the multiple alignment of a proline/glycine-rich TR domain in the *C. elegans *hypothetical protein sequence CE22309 from wormpep173 . 'Positions' denotes the corresponding input sequence index range of this TR domain and 'Copy N' denotes copy number. The consensus error is 0.13 because *nG *= 99, *cG *= 29, *mG *= 583, and *tot *= 1595 (see *Consensus Building *in Appendix). Gap characters are shown in red to emphasize the high indel content of this TR. Below the dashed double line is the consensus sequence followed by the consensus error string shown in blue. Columns of the alignment with 100% character identity have no symbol in the consensus error string. The symbols ':' and '*' denote a column with greater than or equal to 50% character identity and a column with less than 50% character identity respectively.

**Figure 3:**
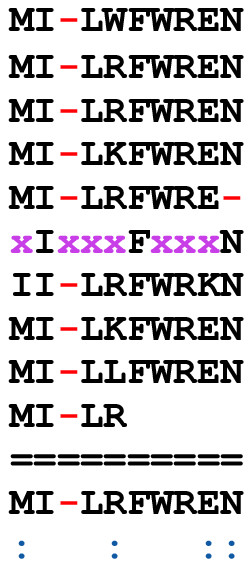
**Discontinuous Domain Merging of TR from *A. thaliana***. Successful merging of non-overlapping TR regions is shown by a TR domain from *A. thaliana *predicted gene product gi 9293925. Characters in the intervening degenerate sequence space that do not match the consensus are each represented by 'x'. This TR has a period of 9, a copy number of 8.67, a consensus error of 0.09 [*nG *= 6, *cG *= 1, *mG *= 9, *tot *= 88 (95-7 x's) (see *Consensus Building *and *Merging *in Appendix)], and is located at sequence positions 1 – 85.

**Figure 4:**
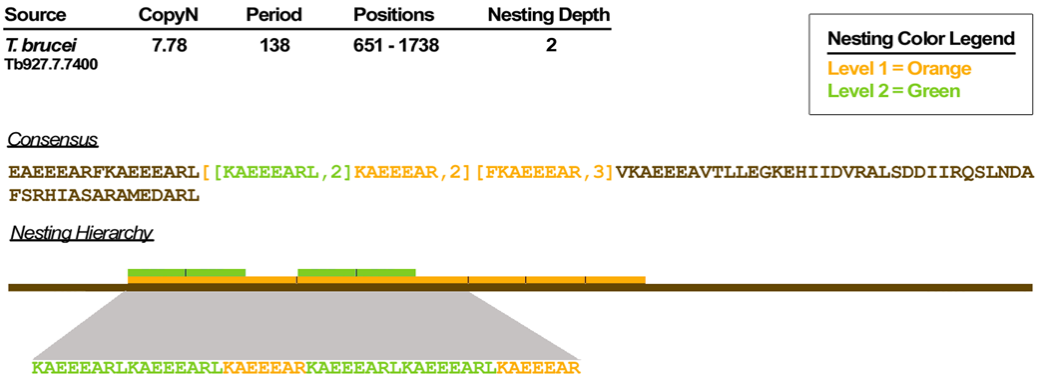
**Example of a Nested TR Architecture**. A nested TR of two hierarchical levels is illustrated with an example from *T. brucei *(copy number = 7.78, period = 138, positions = 651 – 1738). Since a nested TR is by definition, a TR within another TR, the level of nesting depth corresponds to the number of TR domains that encapsulate a particular nested TR. This example shows nested TRs in two representations: the compressed consensus sequence with nested TRs denoted within brackets, and a graphical depiction of the hierarchical structure and distribution of nested TRs, with the consensus represented by the brown bottom bar, and increasing levels of nesting represented by additional bars moving upward.

In addition to efficient TR detection, other important capabilities of XSTREAM are demonstrated with the data shown in Figures 2, 3, 4 and Table 3. A multiple alignment of a degenerate TR domain found in the *C. elegans *hypothetical protein CE22309 is presented in Figure 2. Shown above the alignment are the standard numerical properties reported by XSTREAM for each TR domain: sequence position, period, copy number, and consensus error. Each alignment is additionally described by a consensus sequence (below the dashed double line) and a consensus error string (below the consensus).

**Table 3:** Extreme examples of DNA TRs detected by XSTREAM

**Genomic Sequence**	**Period**	**Copy#**	**Consensus Error**	**Position**
CE Chr III gi 86563600	94	403.6	0.05	7405280–7443237
At Chr I gi 42592260	158	453.7	0.1	14929399–15001291
At Chr I gi 42592260	45653	2.0	0.05	14346314–14437643
	3415	8.5	0.01	12767448–12796444

Anecdotal examples of very high copy number and very long period DNA TRs from chromosome I of *A. thaliana *and chromosome III of *C. elegans *are shown.

The TR example shown in Figure 2 also highlights the utility of the merging feature of XSTREAM when applied to overlapping domains with different periods. Without merging, this TR domain would be reported as several distinct TR fragments. The merging of two non-overlapping TR domains from an *A. thaliana *hypothetical protein (gi 9293925) is illustrated in Figure 3. This example illustrates the utility of incorporating a highly degenerate intervening sequence to define a larger TR domain that, without merging, would have been divided into two discontinuous regions (x's denote non-matching characters). As in proteins, DNA TRs may also contain extensive degeneracy. The high copy number TR domains shown in Table 3 represent additional successful applications of XSTREAM's merging feature. Taken together, the merging of (non)overlapping TR regions allows XSTREAM to successfully model the architectures of TR domains that have accumulated extensive substitution and/or indel mutations, or that have arisen through convergent evolutionary mechanisms.

In addition to extensive degeneracy, TRs may have very long periods and nested architectures. XSTREAM implements a novel long-period filtering procedure (see Appendix) to find TRs with periods ≥1000. The utility of this method is demonstrated by some of the DNA examples in Table 2 and by the long-period *A. thaliana *DNA repeats in Table 3. XSTREAM also incorporates a strategy to find and describe nested TR architectures, represented by the regular expression [*x*,*n*], with *n *denoting the number of tandem copies of substring *x*. An example of TR nesting that shows two levels of nesting is presented in Figure 4. Included in the figure is a block diagram illustrating the hierarchical patterning that epitomizes nested TRs. Taken together, these merging, long-period filtration, and nesting features make XSTREAM a useful tool for detection and architecture modeling of TR domains in both nucleotide and protein sequences.

**Figure 5:**
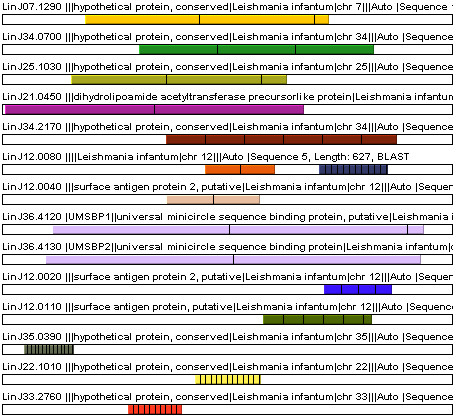
**14 *L. infantum *TR Proteins Found by XSTREAM. **A colored repeat distribution schematic generated by XSTREAM showing 14 *L. infantum *TR-containing proteins found by XSTREAM and not by Goto et al [18]. All protein sequence lengths are normalized, and shown from top to bottom in order of decreasing TR period. TR copies are separated by a vertical black line. Each color corresponds to a specific TR domain. In cases where TR domains of adjacent protein sequences share the same color, such TRs were grouped into the same class by the consensus comparison function (see Appendix).

To validate the utility of XSTREAM for detecting TR-containing proteins, we analyzed the proteomes of five parasite genomes, and compared our output to the TR proteins identified in these same genomes by TRF [18]. Protein sequence datasets for these parasites were downloaded [33] and processed using *minP *= 1, *minD *= 90 and minimum copy number *minC *= 2, or 3. These parameter values were chosen to emulate the TR criteria used in [18] to find TR domains in gene sequences of at least ~250 bp. Setting *minD *= 90 amino acids for XSTREAM corresponds to a slightly more stringent 270 bp minimum. Table 4 summarizes the TRs found by XSTREAM, using *minC *= 3 or *minC *= 2, and by TRF [18]. Using *minC *= 3, XSTREAM identified more TR containing proteins in all parasites except *T. annulata*. In *L. infantum*, the causative agent of Leishmaniasis and the focus of the Goto et al. studies [17,18], XSTREAM found seven TR proteins that they did not identify, while three of the TR proteins found by TRF were not detected by XSTREAM. Upon closer examination of the three "missed" proteins, each was found to have a TR domain with copy number less than 3, which would not be reported by XSTREAM using *minC *= 3. When XSTREAM was rerun with *minC *= 2, all 64 of the previously identified *L. infantum *TR proteins [18] were found, along with 14 additional TR containing proteins that are schematically diagrammed in Figure 5 to illustrate the significant diversity of TR domain architectures within these 14 proteins.

**Table 4:** Number of TR proteins detected in protozoan parasite genomes by XSTREAM and TRF

**Species**	**XSTREAM *MinC *= 3**	**XSTREAM: *MinC *= 2**	**TRF**
*L. infantum*	68 (3, 7)	78 (0, 14)	64
*L. major*	65	74	59
*T. brucei*	115	135	73
*P. falciparum*	252	263	169
*T. annulata*	10	20	11

Numbers in each column represent the number of different TR-containing proteins detected using *minP *= 1 and *minD *= 90 amino acids for XSTREAM, and a minimum score of 500 for TRF. Within the parentheses, the number on the left represents the number of genes identified in [18] that were not identified by XSTREAM and the number on the right represents the number of genes identified by XSTREAM that were not identified by [18]. Comparison of output on an individual protein basis was only possible for *L. infantum *as Goto et al. (2007) did not report identified proteins for the other parasites.

**Figure 6:**
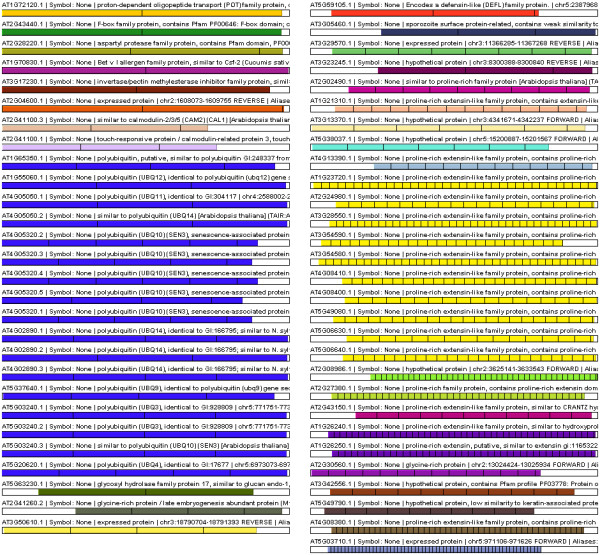
**TR Proteins from *A. thaliana***. A colored repeat distribution schematic generated by XSTREAM showing the 57 TR-containing proteins from *A. thaliana *(TAIR6_pep_20060907) with *minP *= 1 and minimum TR content = 0.7. These protein sequences are ordered by decreasing period from top to bottom. The longest period is shown in the top left panel and the shortest is shown in the bottom right panel. Notice two large classes of protein sequences (polyubiquitins and proline-rich extensin-like family proteins) as determined by grouping their TR domains with the consensus comparison module (see Appendix).

Since TR domains can constitute variable fractions of the parent protein sequence (Figure 5), XSTREAM incorporates the simple concept of *TR Content*, defined as the ratio of the TR domain length to the input sequence length, as an additional metric for comparing modular proteins. Use of this metric allows XSTREAM to filter output using any arbitrary level of TR content, a feature that is illustrated using the protein sequence dataset from *A. thaliana *(TAIR6_pep_20060907). The Arabidopsis proteome was analyzed using parameter values *MinP *= 1 and TR Content ≥ 0.7. The relatively small number of proteins with ≥70% TR content resulting from this analysis are schematically depicted in Figure 6. This output clearly reveals the modular architectures of two large, well-described *A. thaliana *protein families (polyubiquitins with period = 76, and proline-rich extensin-like proteins with period = 25) along with that of additional TR proteins.

## Discussion

The use of *a priori *computational methods to search genome databases for repetitive elements has revealed an abundance of both DNA and peptide repeats in nature, many of which occur in tandemly repeated patterns [6,8,26,27]. The detection and analysis of repeated peptide sequences has received considerable attention in recent years, including the recent publication of a large protein repeats database [26]. Despite the potential importance of such repetitive sequences, the available repeat detection software suffers from both time complexity and output redundancy problems. To address these issues, and to facilitate the detection and modeling of TR structures in general, we developed a new software tool called XSTREAM.

The utility of XSTREAM for efficient and effective detection of degenerate tandem repeats in large input sequence datasets was demonstrated by testing and validation. Practical performance was confirmed by showing that XSTREAM running time can scale linearly with both increasing sequence lengths (up to 202.5 Mbp of DNA sequence) and increasing dataset sizes (up to 230,150 protein sequences). XSTREAM invokes no period limitations and can thus detect TRs with very long periods, as illustrated by the ~45 kbp tandem duplication identified in chromosome I of *A. thaliana *(Table 2). With the implemented merging heuristic, XSTREAM can also identify TR domains with intermittent regions of high degeneracy, such as the TR from *C. elegans *chromosome III with period 94 and copy number >400 (Table 2), and the proline/glycine-rich protein from *C. elegans *shown in Figure 2. In addition, by searching for nested TR structures, XSTREAM detects TRs within TRs (Figure 4), a useful feature for gaining insights into the evolution of complex TR architectures.

Output redundancy is a problem inherent in repeat detection that has often been ignored. For example, using a SW approach, Katti et al. [27] searched Swiss-Prot 38 for TRs with periods between 1 and 20, and compiled the TRIPS database of TRs and their corresponding protein sequence identifiers . In many cases, TRs with different periods were reported that occupy the same protein sequence space. The output of another repeat finding tool [26] also demonstrates the importance of redundancy removal. The ProtRepeatsDB tool  was designed for comparing repeated peptides from many organisms. Though aware of redundancy problems, the strategy implemented by Kalita et al. falls short of providing concise repeat output in numerous cases. For example, ProtRepeatsDB reported 1312 and 568 distinct perfect peptide repeats in the UBQ3 and UBQ12 polyubiquitin sequences from *A. thaliana*, respectively. Unexpectedly, the canonical period 76 TRs known to characterize polyubiquitins were absent. Such highly redundant outputs illustrate the importance of the redundancy removal tactics incorporated into XSTREAM. By invoking several strategies (see Redundancy Elimination in Appendix), including the use of *irreducible *TR periods [24], XSTREAM produces non-redundant TR output. Analysis of the *A. thaliana *proteome by XSTREAM, for example, reports the UBQ3 and UBQ12 sequences only once, with an irreducible, period 76 TR covering virtually the entire protein sequences.

The recent analysis of five protozoan parasite genomes using TRF [18] provided a reasonable reference for testing XSTREAM on genome-scale datasets. Using *minD *= 90 to mimic the TR domain criterion used by Goto et al, XSTREAM detected significantly more TR proteins from all parasite genomes, including all 64 of the previously identified *L. infantum *TR proteins [18]. Further analysis of these 64 TR protein domains revealed that the TR domains identified by both algorithms were comparable in size (data not shown).

## Conclusion

By testing XSTREAM on a variety of sequence data, we demonstrated the utility of this new genome data-mining tool for identifying TRs with diverse periods and domain sizes, varied levels of degeneracy, and complex architectures. These capabilities should facilitate potentially significant applications. For example, TRs present in parasitic pathogens are known to elicit important immunological responses that may provide antigenic protection (e.g., [19]). New computational approaches for detecting TR proteins might thus be useful for identifying novel protein antigens useful for diagnostics and vaccine development [17,18]. Secondly, since TR domains are characteristic of modular structural proteins, use of XSTREAM may lead to the *in silico *discovery of phylogenetically diverse proteins with novel biomaterials and biomimetic applications.

## Availability and requirements

Project Name: XSTREAM

Project home page and availability: 

Operating system(s): Platform independent

Programming language: Java

Any restrictions to use by non-academics: yes, contact author JBC for details

## List of abbreviations used

TR, tandem repeat; TRF, Tandem Repeats Finder [29]; DP, dynamic programming; GRDP, gap-restricted dynamic programming; SSA, sequence self-alignment; SW, sliding window; SE, seed extension; WDP, wrap-around dynamic programming; CC, consensus comparison; ET, edge trimming; CW, comparison wobble; *minP*, minimum period; *minC*, minimum copy number; *minD*, minimum TR domain length; HPS, heuristic partitioning strategy

## Competing interests

The author(s) declares that there are no competing interests.

## Authors' contributions

AMN conceived of, designed, implemented, tested, and validated XSTREAM, and wrote the manuscript. JBC conceived of, tested, and validated XSTREAM, and wrote the manuscript. Both authors approved the final manuscript.

## Appendix

### Preliminary Notations

• *S *= input sequence, which takes values from alphabet {A,C,G,T} for nucleotide sequences and alphabet {A,C,D,E,F,G,H,I,K,L,M,N,P,Q,R,S,T,V,W,Y} for proteins

· |*S*| = length of *S*

· *S*[*j*] = the character at index *j *in *S *with *j *≥ 0

· *S*[*i*, *j*] = the subsequence in *S *from index *i *to index *j *inclusively

• *Xi *= TR domain *i*

· |*Xi*| = length of entire TR domain *Xi*

· *Xi*[*j*] = repeat copy *j *in *Xi *with *j *≥ 0

· |*Xi*[*j*]| = length of copy *j*

· |*Xi*[]| = size of array *Xi*[]

· *XiS *= lowest index of *Xi*; starting position in *S*

· *XiE *= highest index of *Xi*; ending position in *S*

· *XiSE *= index range [*XiS*, *XiE*]

· *Ei *= copy number (exponent) of *Xi*

· *Ci *= consensus sequence of *Xi*

· *Pi *= period of *Xi *= period of *Ci*

· *CEi *= consensus error of *Xi *=

- *Without gaps: *# of mismatching characters to consensus/total # of characters in aligned *Xi*

- *With gaps: *see *Consensus Building*

· *Ii *= indel error of *Xi *= # of gaps in aligned *Xi*/total # of characters in aligned *Xi*

· *Ri *= referential repeat copy of *Xi*: used during TR domain expansion and maximality

• {*X*} = {*X*_0_, *X*_1_,..., *X*_*n*_} = set of all identified TR domains

### Pre-Processing

To find repeats of various periods in any FASTA-formatted input sequence *S*, XSTREAM looks, by default, for exact repetitions (seeds) of lengths 3 and 5. Length 7 is also used if |*S*| ≥ 2000. Seed lengths are user-adjustable. XSTREAM records the distance between each pair of adjacent seeds, |*p *- *q*|, where the lowest index in *S *of each seed in the pair is represented by *p *and *q *respectively, and *p *<*q*. All seed positions and distances between adjacent seeds are stored and accessed using a hash table. In addition, XSTREAM records in an integer array *M, *the hashcodes and sequence indices for all seeds of minimum length *L*, where *L *= 3 by default. For instance, a seed of length *L *starting in position 5 in *S *would have its hashcode stored in *M*[5]. The utility of *M *is explained shortly.

### TR Detection

#### Seed Extension

XSTREAM traverses the distance list in order of increasing distance, and for each set of identical distances, moves down *S *in order of increasing indices. For a given seed pair, let *p*, *q *be defined the same as previously and let *x*, *y *be the starting positions of two sequence iterators, where *x *= *p *+ *L*, *y *= *q *+ *L*. Further, let *d *= |*p *- *q*|, p* = *p *+ *d *- 1, and q* = *q *+ *d *+ *ε *- 1 where *0 *≤ *ε *≤ *g *(for explanation of *g*, see Gap-Restricted Dynamic Programming below; ε is explained shortly) and q* < |*S*|. Because the seeds of each matching pair are of length *L*, *x *and *y *iterate through *S *in the regions *S*[*p *+ *L*, p*] and *S*[*q *+ *L*, q*]. Note that in the case *L *= 3, the minimum copy number is 2 for all periods except periods 1 and 2, which cannot have copy number less than 4 and 2.5 respectively. We now refer to array *M*, which was constructed during seed detection. To bypass individual character comparison, *M *is interrogated for matching hashcodes. If *M*[*x*] = *M*[*y*] and (*x *+ *L*) = p* and (*y *+ *L*) ≤ q*, *x *and *y *are incremented by *L *(since each hashcode in *M *corresponds to a repeat of length *L*), and a match of *L *characters is recorded. By comparing hashcodes instead of substrings and by allowing jumping in blocks of *L *characters, usage of *M *can decrease XSTREAM running time. If *M*[*x*] = *M*[*y*] and *x *≤ p* < (*x *+ *L*), a match of length *min*(*L*, p* - (*x *- 1)) is recorded, and SE terminates. If *M*[*x*] ≠ *M*[*y*] and *g *= 0, XSTREAM compares the character pair in *S *at *S*[*x*] and *S*[*y*]. Whether or not *S*[*x*] = *S*[*y*], if (*x *+ 1) ≤ p* and (*y *+ 1) = q*, *x *and *y *are incremented by 1, and XSTREAM returns to hashcode comparison using *M*.

If the case arises where *M*[*x*] ≠ *M*[*y*] and *g *> 0, a novel procedure termed "comparison wobble" (CW) is invoked. CW allows for efficient approximation of indels using array *M *and parameter *g*. This procedure is one-sided, in that it fixes *x *and allows for variations in *y*, denoted by y*. We place the following restrictions on y*:

i) |y* - *y*| ≤ *g*

ii) y* < |*S*|

iii) If y* <*y*, then (*y *- y*) ≤ *L *AND (*y *- y*) <*d*. We enforce this constraint to avoid comparing subsequences at the same pair of positions in *S *more than once.

iv) y* > Ω, where Ω = highest index in *S*[*q *+ *L*, q*] with matching character from the current seed extension – e.g. if last match was *M*[15], then Ω = 15 + *L *- 1; if last match was *S*[15], then Ω = 15. This rule prohibits matching redundancy.

**Figure 7:**
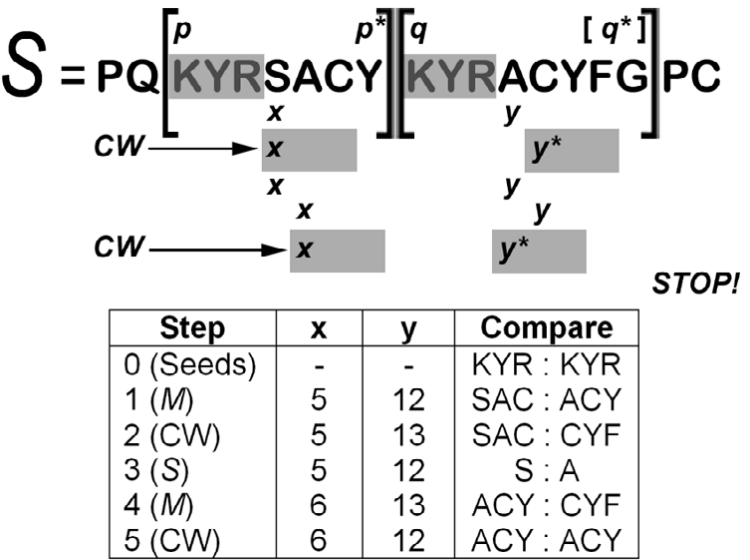
**Seed Extension Example**. Extension of the seed pair 'KYR' is illustrated using the input sequence *S *= PQKYRSACYKYRACYFG (|*S*| = 19) with parameter values *L *= 3 and *g *= 1. A tracing of this SE example is shown for the sequence iterator values (*x*, *y*) and the compared subwords in *S*. The SE subroutine used in each step is indicated in parentheses, where *M *= hashcode array and CW = consensus wobble.

If ∃y* such that M[*x*] = *M*[y*], XSTREAM records a match of *min*(*L*, p* - (*x *- 1)), increments *x *by *L*, sets *y *← (y* + *L*), and if *x *≤ p*, returns to standard SE (see above paragraph). Because *y *← (y* + *L*), it is possible that *y *moves beyond *q *+ *d *- 1, hence the need for *ε*. In addition, if a match is found when y* <*y *(prior to updating *y*), the mismatch record is adjusted to take into account any currently matching characters that were initially found to be non-matching. If *M*[*x*] ≠ *M*[∀y*], XSTREAM transitions to single character comparison using *S*, and then if space permits, returns to standard comparison using *M*. An example of seed extension with CW is shown in Figure 7.

#### TR Domain Expansion

Seed extension operates on seed pairs, and therefore, if successful, only yields putative TRs of copy number 2. To further extend each potential TR *Xi*, XSTREAM implements two procedures, although the second one is used only if *g *> 0. First, *x *is reset to *p*. In this way the copy in *Xi *with the lowest index serves as the character comparison reference repeat *Ri*. The value given to *q *depends upon whether XSTREAM is attempting to extend *Xi *downstream or upstream of *Xi*'s current sequence region. If downstream, *q *is incremented by *d*. If upstream, *q *is initially set to *p *- *d*, and decremented by *d *thereafter. The first method for domain expansion is exactly the same as seed extension except *x *= *p*, *y *= *q*, and the evaluated regions in *S *are *S*[*p*, p*] and *S*[*q*, q*], where 0 ≤ *q *≤ (|*S*| - *d*). If this procedure is successful, the new copy is added to *Xi*. If unsuccessful and if *g *> 0, XSTREAM invokes the second procedure, which uses GRDP (see Gap Restricted Dynamic Programming below) on the same regions in *S*. GRDP is better, albeit slower, than CW at identifying indel regions. Upon completion of GRDP, the number of matching characters in the alignment is determined and if that number is high enough, the new copy is added to *Xi*. Following success by either expansion method, *q *is updated and domain expansion is performed again. If *i *is not satisfied, domain expansion ceases, and the current candidate TR domain is sent to the maximality function.

#### Maximality

The maximality procedure makes use of *Ri*, with *p *remaining equal to the lowest index of *Ri*. This method finds the longest valid prefix and suffix of *Ri *by searching downstream and upstream of *Xi *respectively. A DP sequence alignment scoring scheme is used, with match = 2, mismatch = -4, and gaps = -4 (user modifiable). Let *l *= *XiS*, *r *= *XiE*, *left *= *l *- *min*(*Pi*, *l*), and *right *= *r *+ *min*(*Pi*, |*S*| - (*r *+ 1)). Further, let *Q*_1 _= *S*[*left*, *l *- 1], *Q*_2 _= *S*[*r *+ 1, *right*], *RiQ*_1 _= *S*[(*p *+ *Pi*) - *min*(*Pi*, *l*), *p *+ *Pi *- 1], and *RiQ*_2 _= *S*[*p*, *p *+ *min*(*Pi*, |*S*| - (*r *+ 1)) - 1]. Since XSTREAM needs to find the character pair that corresponds to the highest score, it reverses the order of characters for both *Q*_1 _and *RiQ*_1 _prior to alignment. If *g *> 0, GRDP is used to align *Q*_1 _with *RiQ*_1 _and *Q*_2 _with *RiQ*_2_. If *g *= 0, the sequences are aligned so that the members of each sequence pair overlap 100%. XSTREAM uses the DP scoring scheme regardless of whether GRDP is used. The highest scoring indices in *Q*_1_, *Q*_2 _are denoted *Q*_1_* and *Q*_2_* respectively. If, at index *Q*_1_*, the score exceeds 0, *Xi *is extended upstream by (*Q*_1_* + 1) characters, and if the score for index *Q*_2_* is greater than 0, *Xi *is extended downstream by (*Q*_2_* + 1) characters.

#### Copy Number Computation

For a given *Xi*, using the indicator function I (I[true] = 1; I[false] = 0):

•Ei=∑∀j ∈XiI[|Xi[j]|≥Pi]+I[|Xi[j]|<Pi]⋅(|Xi[j]|/Pi) 
 MathType@MTEF@5@5@+=feaafiart1ev1aaatCvAUfKttLearuWrP9MDH5MBPbIqV92AaeXatLxBI9gBaebbnrfifHhDYfgasaacH8akY=wiFfYdH8Gipec8Eeeu0xXdbba9frFj0=OqFfea0dXdd9vqai=hGuQ8kuc9pgc9s8qqaq=dirpe0xb9q8qiLsFr0=vr0=vr0dc8meaabaqaciaacaGaaeqabaqabeGadaaakeaacqWGfbqrcqWGPbqAcqGH9aqpdaaeqbqaaiabbMeajjabbUfaBjabbYha8jabdIfayjabdMgaPjabbUfaBjabdQgaQjabb2faDjabbYha8jabgwMiZkabdcfaqjabdMgaPjabb2faDjabgUcaRiabbMeajjabbUfaBjabbYha8jabdIfayjabdMgaPjabbUfaBjabdQgaQjabb2faDjabbYha8jabgYda8iabdcfaqjabdMgaPjabb2faDjabgwSixlabbIcaOiabbYha8jabdIfayjabdMgaPjabbUfaBjabdQgaQjabb2faDjabbYha8jabb+caViabdcfaqjabdMgaPjabbMcaPiabbccaGaWcbaGaeyiaIiIaemOAaOMaeeiiaaIaeyicI4SaemiwaGLaemyAaKgabeqdcqGHris5aaaa@6DA2@

Computing *Ei *in this way demands that *Ei *≤ |*Xi*[]|. Both gap and masked ('x', see Merging) characters are not considered during *copy number computation*. Ei is updated whenever XSTREAM changes *XiS*, *XiE*, *Pi*, or *Xi*'s multiple alignment.

#### Sequence Masking

After each successful seed extension, XSTREAM masks the sequence space corresponding to the newly detected TR domain in order to reduce both running time and repeat redundancy (see Redundancy Elimination I and Two-stage TR Detection below). Afterward, the next seed pair, if one exists, is extended.

#### Period Offset

If *g *> 0 and comparison wobble is successfully used, then the period *Pi *for a given TR *Xi *may need adjustment. To approximate a better period, Pi*, we turn to the offset y* - *y *for every CW success for a given *Xi*. Let *So *= Σ(y* - *y*), for all successful extensions, i.e. *Xi*[∀*j*] ≠ *Ri*. Then, Pi* = *Pi *+ (*So*/*Ei*), and *Pi *← Pi*. Therefore, *Pi *is updated using the average period offset. This function is important for TR domain parsing when *g *> 0, since *Pi *is used to derive a temporary *Ci*, which is needed for TR domain alignment.

### TR Characterization

#### TR Domain Parsing

In order to best characterize any TR domain *Xi*, its copies are aligned to one another and used to create a consensus sequence *Ci*. We describe our consensus derivation procedure shortly. To align *Xi*, it must be partitioned into its repetitive parts. For the case *g *= 0, starting from *XiS*, *S*[*XiS*, *XiE*] is cut into as many tandem fragments of length *Pi *as possible. Because of maximality, *Xi*'s last copy may have length less than *Pi*. Multiple alignment of *Xi *is achieved by simply stacking all copies in the order they occur in *S*. If *g *> 0, partitioning of *Xi *is much more complex. To preserve practical running time for the case *g *> 0, we use one of two segmentation tactics. Both methods require a putative consensus sequence *Ci *for a given *Xi*. XSTREAM therefore initially partitions *Xi *in the same way as when *g *= 0. Afterward, *Xi *is aligned using a multiple alignment algorithm that we describe shortly. Following alignment, a transient *Ci *is derived. We now compare/contrast XSTREAM's two partitioning procedures for the case *g *> 0.

WDP can optimally parse a TR domain *Xi *in O(*mn*) time given a representative copy of length *m *(i.e. *Ci*), where *m *= *Pi *and *n *= |*Xi*| [31]. This time complexity is practical up until *mn *is very large. Since XSTREAM has no period limitations, we developed a heuristic partitioning strategy (HPS) that uses GRDP. When *mn *> 1,000,000 and *m *> *g*, XSTREAM invokes HPS; otherwise, WDP is used. Our version of WDP requires two passes through the DP matrix and therefore computes 2 *mn *scores, whereas GRDP computes < (2*g + *1)*n *scores. To ensure that HPS makes less DP matrix computations than WDP, we require *m *> (*g *+ 1/2), which is equivalent to *m *> *g *since *m*, *g *only take integer values.

As mentioned, both partitioning strategies require *Ci*. WDP aligns *Ci *to the domain *D *= *S*[*XiS*, *XiE*]. Afterward, *D *is cut between every adjacent instance of *Ci*. HPS works by first building a concatamer of *Ci *comprised of *n *copies of *Ci*, where *n *= |*Xi*|/|*Ci*|. Because *n *may take a non-integer value, the consensus concatamer can have more or fewer copies than an optimal partitioning of *Xi*. After pairwise alignment to *D *using GRDP, |*Xi*| is segmented in the same way as described for WDP.

#### Multiple Alignment

XSTREAM employs the STAR alignment algorithm for multiple sequence alignment. The center sequence is computed using GRDP exclusively. We elected to use GRDP over standard DP because the number of pairwise alignments that are needed increases as a function of (*floor*(*Ei*))^2 ^(we use the floor function since *Ei *may be non-integer), in which case the last copy is excluded from being a center sequence. Because our version of STAR does not use standard DP, it will not always compute an optimal center sequence. Nevertheless, to maximize the practicality of XSTREAM for large dataset analyses, we decided that the order of magnitude performance gain provided by GRDP outweighs the possible decrease in multiple alignment quality. Since GRDP requires input sequences of the same length, we temporarily replicate *Xi*, denoted by Xi*, and add the dash character '-' to the rightmost end of all copies of Xi* where |Xi*[*j*]| <*max*(|*Xi*[∀*j*]|) until |Xi*[∀*j*]| = *max*(|*Xi*[∀*j*]|). We then find the center using Xi*. Following center sequence determination, the TR multiple alignment is constructed using the conventional STAR alignment strategy. Because practical running time is emphasized in our implementation, pairwise sequence comparisons during STAR Alignment may be computed in a non-optimal manner using GRDP.

#### Consensus Building

XSTREAM's consensus derivation procedure makes use of the majority rule. That is, for the multiple alignment of a given *Xi*, the majority character in each column of the alignment is selected. If no majority exists, then, by and large, the topmost character is chosen. However, if |*Xi*[]| = 2, and if within a given column, one character is a gap and the other is a non-gap, the gap character is added to the consensus. If, on the other hand, |*Xi*[]| > 2, and if within a column, a gap character is tied in number with one or more non-gap characters, the topmost non-gap character is added to the consensus.

To compute the consensus error *CEi *for a given *Xi*, we keep track of four variables:

i) The non-gap counter, denoted *nG*, tallies every non-gap character that does not match its corresponding consensus character.

ii) The majority gap counter, *mG*, records the number of gaps in all columns where the majority character is a gap.

iii) A user-modifiable constant, g* (=3, by default), specifies the maximum number of consecutive gaps in an alignment row that can be counted toward *CEi*. For each row of the alignment, we count the number of successive gaps that do not match the consensus until either that number equals g*, a non-gap character is reached, or the consensus contains a gap. We resume counting gaps the next time a gap is encountered in a column where the consensus character is a non-gap. Let *cG *equal the final count.

iv) Let *tot *= total number of characters in the multiple alignment of *Xi*, including gaps.

We set *CEi *= (*nG *+ *cG*)/(*tot *- *mG*). The quantity *mG *is subtracted from *tot *so that gaps in columns with a gap majority do not decrease *CEi*. Further, the addition of *cG *to the numerator functions to limit the extent to which gaps increase *CEi*. We dampen the role gaps play in *CEi *since they are artificial characters. In addition, we force *Pi *to equal the number of non-gap characters in *Ci*, and therefore, if necessary, *Pi *is updated.

#### Edge Trimming

For each *Xi*, *Edge Trimming *(ET) moves downstream from *XiS *and upstream from *XiE*, deleting characters that mismatch with *Ci *until the first matching character pair is found from each direction. *Xi *is realigned if truncation is successful from the top-left, since otherwise we would start the alignment with one or more gaps. If ET is only successful from the bottom right, no realignment is necessary. In this case, XSTREAM removes both the flagged bottom right portion of the alignment as well as any columns that contain all gaps. If ET is a success from either direction, *Ci *is rebuilt. For each *Xi*, ET is iteratively invoked until either |*Xi*[]| = 2 or both edges of *Xi *agree with *Ci*.

### Post-Processing

#### Merging

XSTREAM iterates through {*X*} in order of increasing period. Given *Pi*, ∀*i *∈ {*X*}, the following routine is executed:

(1) Define *Xtra *as *min*(2*Pi *- 1, *Pi *+ *min*(*σ*, *g *+ (1 - *i*)·*Pi*), *max*(*Pj*) ∀*j *∈ {*X*}), where by default, *σ *= 50. *Xtra *dictates the breadth of periods from which to draw TRs for merging. The conditions restricting *Xtra *were chosen to avoid messy and insensible TR domain characterizations as well as to maintain practical running time.

(2) Let TR set {*B*} = *Xj*, ∀*j *∈ {*X*}, where *i *≠ *j *and *Pi *≤ *Pj *≤ *Xtra*. Set {*X*} ← ({*X*} - {*B*}). Note: from step (3) to step (10), we only refer to TRs from {*B*}.

(3) Sort {*B*} in increasing order of *XjS*, ∀*j *∈ {*B*}.

(4) Starting with *m *= 0, we examine *Xm *and *Xn*, ∀*m*, *n *∈ {*B*}, where *n *= *m *+ 1

(5) Let *Q *denote the maximum allowable sequence space between two combinable TRs, and set *Q *= *min*(*μ*_1_, *μ*_2_·*Pm*)·*Pm*. By default, *μ*_1 _= 10 and *μ*_2 _= 0.25.

(6) **if **|*XmSE *∩ *XnSE*| ≠ Ø **or **0 < (*XnS *- *XmE*) ≤ *Q*, compute similarity *s *of *Cm *and *Cn *using the consensus comparison function (refer to consensus comparison section).

**else **go to step (11).

(7) **if ***s *≥ *i*, merge *Xm *and *Xn*.

**else **go to step (11).

(8) **if **|*XmSE *∩ *XnSE*| ≠ Ø, perform the following procedure: From step (6) we obtained the index *CnP *(refer to consensus comparison section) corresponding to the best cyclical permutation of *Cn *when aligned to *Cm*. We repartition *Xn *by slicing its alignment vertically at *CnP*, thus ensuring *Xn *is in phase with *Xm *before consolidation. We then merge *Xm *and *Xn*, forming *Xmn *= (*Xm *∪ *Xn *- *Xm *∩ *Xn*). Go to step (10).

(9) **if **|*XmSE *∩ *XnSE*| = Ø, perform the same procedure as in step (8) with the exception that the sequence space between *Xm *and *Xn *must be incorporated into *Xmn*:

i) Let *z *equal the index in *S *that corresponds to the character in *Xn*[0] that is in the same alignment column as *CnP*. Let sequence *k *= *S*[*XmE *+ 1, *z *- 1].

ii) Add *Xm *in its original form to *Xmn*.

iii) Tile *k *in accordance with *Cm*. To do this, cut *k *into as many consecutive fragments {*f*} of length *Pm *as possible. Start cutting *k *from the end with the lowest index.

iv) Given *fi*, ∀*i *∈ {*f*} (tile fragments in order of increasing indices in *k*),

**if **|*fi*| = *Pm*, use the consensus comparison module to compute similarity *s *of *fi *and *Cm*.

**if ***s *<*η*, where *η *<*i *and *η *= .5 by default, replace all characters in *fi *that do not match to *Cm *with 'x' and add *fi *to *Xmn*.

**else **cut *fi *at the index corresponding to its best cyclical permutation, resulting in *fi*_1 _and *fi*_2_.

**if **(|*Xmn*[*max*(*j*)]| + |*fi*_1_|) ≤ (*g *+ *Pm*), append *fi*_1 _to *Xmn*'s last row.

**else ***fi*_1 _becomes a new row in *Xmn*.

Regardless of what happens to *fi*_1_, since *fi*_2 _is in phase with *Cm*, *fi*_2 _becomes a new row in *Xmn*.

**else if **|*fi*| <*Pm*, add *fi *to *Xmn *in the same manner as *fi*_1 _(above).

v) Following the incorporation of *k*, add *Xn *to *Xmn *in the same way as in step (8).

(10) Remove all gap characters from *Xmn*, perform multiple alignment on *Xmn *(without parsing) and derive consensus. We do not include the 'x' character (see (9 *iv*)) in the calculations of *Emn*, *Cemn *and *Imn*. **if ***Xmn *meets TR retention criteria, set *Xm *← *Xmn *and {*B*} ← {*B*} - *Xn*.

(11) **if ***m *< |*B*| - 2, increment *m *by [0 if merging successful; 1 otherwise] and go to step (4).

**else **set {*X*} ← {*X*} ∪ {*B*}.

#### Finishing Touches

The following TR domain refinement procedures are invoked in the order presented:

(1) Maximality – Rerun the maximality function on each *Xi*, but set *Ri *← *Ci*. We invoke maximality again because using *Ci *as a reference copy may allow for additional expansion of *Xi*.

(2) Realignment – For each TR in {*X*}, make a copy of *Xi*, denoted Xi*, and perform multiple alignment on Xi* using *Ci *as the center sequence. *Ci *is not included in the final alignment of Xi*. If CEi* <*CEi*, we set *Xi *← Xi*.

(3) Reducibility – Rerun redundancy elimination procedure II (see below) on every realigned TR in {*X*}.

(4) Overlap Removal – If allowed by user, send {*X*} to redundancy elimination algorithm III (see below).

(5) Nesting – By default, send {*X*} to nesting procedure (see below).

#### Consensus Comparison

For clustering different TRs, we compare their consensus sequences. In order to effectively compare consensus sequences we take into account TR phase variation – the same TR can have different starting points, leading to consensus sequences of different phases. More formally, every irreducible *Xi *can occur in *Pi *cyclical permutations, and if a given TR *Xj *has *Ej *≥ 2 + (*Pj *- 1)/*Pj*, then *Xj *has *Pj *valid consensus sequence phases. Therefore, we must evaluate up to *Pi *consensus alignments for every pair of TRs with period *Pi *that also satisfies the same copy number condition as *Xj*. For simplification, we treat all TRs the same, regardless of copy number. Given a pair of TRs, *Xi *and *Xj *where *Pi *= *Pj*, XSTREAM fixes *Ci *and aligns as many phases of *Cj *to *Ci *as are needed to establish similarity. If *Consensus Comparison *is called from the merging procedure, all phases of *Cj *are aligned to *Ci *to locate the best-aligned cyclical permutation. The leftmost character in the highest scoring phase of *Cj*, denoted by *CjP*, is used during TR merging. Otherwise, only sufficient similarity is needed, and thus XSTREAM may align less than all phases of *Cj*. If *g *> 0, all gaps are removed from *Ci*, *Cj *prior to alignment. All alignments of *Ci, Cj *are computed using GRDP. For each alignment, XSTREAM counts the number of matching characters and stores the highest match count so far in *N*. If *N*/*Pi *≤ *i*, XSTREAM groups *Xi *and *Xj*. The time complexity of comparing *Ci *and *Cj *is O(*Pi*^2^) because of *Pi *alignments and O(*Pi*) alignment time. For every newly established TR group, the consensus sequence with the lowest index in {*X*} becomes the group head or referential consensus, and is used for all subsequent comparisons. The time complexity for performing all consensus comparisons of the same period without considering alignment time is O(|*X*|^2^). Therefore, the total time complexity of *Consensus Comparison *is O(|*X*|^2^*Pi*^2^).

### Gap-Restricted Dynamic Programming

A major obstacle to efficient alignment of gapped TRs is dynamic programming (DP), which, for global pairwise-sequence alignment, has time complexity O(*n*^2^), where *n *= TR period. Because optimal alignment of TR copies may, in some cases, place a temporal burden on the user, we explored heuristic options. We decided to implement a non-optimal variant of pairwise global sequence alignment DP, which we call gap-restricted DP (GRDP). GRDP requires a user-modifiable parameter, *g*, which governs the maximum number of consecutive gaps that can be used during GRDP pairwise alignment. Because of *g*, the maximum traceable width of the DP matrix is held constant for all periods, is equal to 2*g *+ 1, and is symmetrically distributed with respect to the main diagonal. As a result, GRDP has space complexity θ(*n*) and time complexity θ(*n*), enabling a 1:1 correspondence between increasing period and running time. The following recursion describes GRDP:

score(i,j)=max⁡{score(i−1,j)+gapscore(i,j−1)+θscore(i+1,j−1)+gap
 MathType@MTEF@5@5@+=feaafiart1ev1aaatCvAUfKttLearuWrP9MDH5MBPbIqV92AaeXatLxBI9gBaebbnrfifHhDYfgasaacH8akY=wiFfYdH8Gipec8Eeeu0xXdbba9frFj0=OqFfea0dXdd9vqai=hGuQ8kuc9pgc9s8qqaq=dirpe0xb9q8qiLsFr0=vr0=vr0dc8meaabaqaciaacaGaaeqabaqabeGadaaakeaacqWGZbWCcqWGJbWycqWGVbWBcqWGYbGCcqWGLbqzcqGGOaakcqWGPbqAcqGGSaalcqWGQbGAcqGGPaqkcqGH9aqpcyGGTbqBcqGGHbqycqGG4baEdaGabaqaauaabaqadeaaaeaacqWGZbWCcqWGJbWycqWGVbWBcqWGYbGCcqWGLbqzcqGGOaakcqWGPbqAcqGHsislcqaIXaqmcqGGSaalcqWGQbGAcqGGPaqkcqGHRaWkcqWGNbWzcqWGHbqycqWGWbaCaeaacqWGZbWCcqWGJbWycqWGVbWBcqWGYbGCcqWGLbqzcqGGOaakcqWGPbqAcqGGSaalcqWGQbGAcqGHsislcqaIXaqmcqGGPaqkcqGHRaWkiiGacqWF4oqCaeaacqWGZbWCcqWGJbWycqWGVbWBcqWGYbGCcqWGLbqzcqGGOaakcqWGPbqAcqGHRaWkcqaIXaqmcqGGSaalcqWGQbGAcqGHsislcqaIXaqmcqGGPaqkcqGHRaWkcqWGNbWzcqWGHbqycqWGWbaCaaaacaGL7baaaaa@779E@

**Figure 8:**
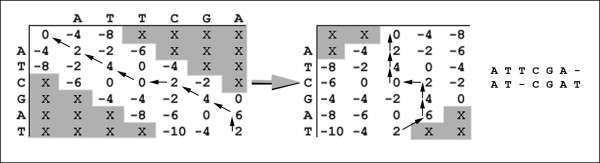
**Sequence Alignment using GRDP**. The matrix on the left represents GRDP sequence alignment of sequences 'ATTCGA' and 'ATCGAT' with *g *= 2 and space complexity O(*n*^2^). Since *g *places an upper bound on traceable matrix width, we only use O(*n*) space, as shown with the matrix on the right. Notice that because the width of the matrix on the right is 2 *g *+ 1, it accommodates all of the relevant information from the matrix on the left. The resulting pairwise alignment is also shown.

Note that depending on *g*, we place constraints on where each score possibility can be computed. The parameters *gap *and *θ *denote gap penalty and match/mismatch values respectively. By default, all DP procedures use values *gap *= -4, *mismatch *= -4, and *match *= 2. An example of GRDP alignment is shown in Figure 8. Also, note that if *g *= 0, XSTREAM completely disallows gaps, and thus the decision to allow insertions/deletions (indels) is left up to the user. By default, *g *= 3. In addition, our implementation of GRDP requires input sequences of equal length. In cases where input sequences have different lengths, both sequences are made the same length by appending gap characters to the shorter sequence. Any columns with two gaps in the resulting pairwise alignment are removed. Since standard DP is practical in many situations, several functions of XSTREAM toggle GRDP on and off depending on projections of time complexity. GRDP is used in four major functions: TR domain expansion, TR parsing, multiple alignment, and consensus comparison.

### Redundancy Elimination

XSTREAM implements three strategies to eliminate two types of TR redundancy – reducible TR periods and TR domain overlap.

I) XSTREAM searches for TRs in order of increasing period. As TRs are found, their corresponding sequence space is flagged, preventing further searching in processed sequence regions. This tactic combats both kinds of redundancy and reduces running time.

II) To combat reducible TR periods, XSTREAM is rerun on the consensus sequence of each TR domain from the input sequence. (see Figure 1) If the consensus sequence *Ci *of *Xi *contains a TR domain *xi *that spans *Ci*'s entire length, XSTREAM repartitions *Xi *using the consensus of *xi*, resulting in Xi*, whose period is an even multiple of *Xi*'s period. Xi* is retained and *Xi *erased (Xi*→*Xi*) if Xi* passes the user-adjustable TR filtration criteria.

III) The following redundancy elimination method, invoked by default, functions to remove TR domain overlap. The user can control the execution and parameters of this method because it may not always be desirable to remove TR domain overlap and because we are convinced that the amount of reasonable overlap among TR domains is an arbitrary matter. We now state the rules that determine whether for a given TR pair *Xi *and *Xj*, XSTREAM deletes one or neither. The rules are enforced in the order they are presented; i.e. rule set (i) must fail to move to rule set (ii) and so on. Let *I *= |*XiSE *∩ *XjSE| *(length of intersection of TR domains *i*, *j*). By default, *α *= .9, *β *= .75, *γ *= .9, and *δ *= .6.

i) **if **(*α*·|*Xi*|) ≤ *I *≤ |*Xi*| **and **|*Xi*| ≤ |*Xj*|

   **if **|*Xi*| < (*β*·|*Xj*|)

      delete *Xi*

   **else if **|*Xi*| < (*γ*·*|Xj*|) **and **(*Ei *<*Ej ***or ***CEi *> *CEj*)

         delete *Xi*

      **else if **|*Xi*| ≥ (*γ*·*|Xj*|) **and ***Ei *<*Ej*

            delete *Xi*

         **else **delete *Xj*

ii) Same as (i) but swap *i *and *j*

iii) **if ***I *≥ (*δ*·*max*(|*Xi*|, |*Xj*|))

   **if ***CEi *≥ *CEj ***and ***Ei *≤ *Ej*

      delete *Xi*

   **else **delete *Xj*

iv) **if ***I *≥ (*δ*·*min*(|*Xi*|, |*Xj*|))

   delete *min*(|*Xi*|, |*Xj*|)

### Two-Stage TR Detection

As shown in Table 1, XSTREAM allows the user to restrict the TR period range. If *MinP *<*T *and *MaxP *≥ *T*, TR detection proceeds in two phases, where phase I examines periods = *T*, and phase II examines periods <*T*. By default, *T *= 10. This procedure reduces the frequency of inconsistent results. We now describe our reasoning.

As mentioned in Redundancy Elimination I, TRs are identified in order of increasing period and sequence space is masked for every successful seed extension. Because of these two facts and because the value of *MinP *can be altered, it is possible to differentially characterize the same TR domain *Xi*, or perhaps miss *Xi *altogether, for the case *Pi *≥ *max*[all tested *MinP *values]. This problem can occur because as XSTREAM moves up the period ladder toward *Pi*, different stretches of sequence space may be removed in and around *Xi *for different values of *Min*. We determined empirically that by first scanning upward from a short period, such as 10, we could greatly mitigate this problem. To illustrate, see Figure 2 for an example of a TR domain containing many short period TRs. Without Two-Stage TR Detection, this period 152 TR domain would not be reported since most of its sequence space would be masked by its constituent TRs.

Following completion of phase I, all masked sequence space is reset to unused, thereby allowing shorter period TRs to be found independently of longer period TRs. Redundancy removal strategies II and III are invoked later and will remove any redundancy caused by XSTREAM's two-stage TR detection procedure.

### Long Period TR Filter

To ensure pragmatic running time for all possible periods, we implemented a heuristic that governs seed extension for periods greater than or equal to 1000 characters. If |*S| *≥ 2000, during seed detection, an additional hashcode array M* is kept, which stores hashcodes and sequence positions for seeds of maximum length L*, which by default is 7. Then, for every pair of seeds with distance ≥ 1000, XSTREAM initially invokes a filtration step, which jumps across M* a user-defined number of times *t *and looks for matching hashcodes. This method is identical to seed extension as described earlier, except that *S *is not used and *x *is incremented by *floor*(*d*/*t*) after each hashcode comparison. Thus, if *g *> 0, CW can be invoked. XSTREAM runs standard seed extension and TR domain expansion (using M* and L*) on periods ≥ 1000 if and only if t* matches are recorded during the filtering phase, where t* = *t*/3. Therefore, seed pairs with distances ≥ 1000 are subjected to a quick and preliminary filter, which although imperfect, drastically reduces running time for input sequences on the chromosome size scale. By default, *t *= 20.

### Nesting

Within each TR consensus sequence, XSTREAM searches for nested TRs – TRs that occur within TRs. This is a novel feature in the domain of protein analysis software and may provide important information about primary sequence architectures and peptide TR evolution. For a given *Xi*, we define a nested TR as a TR present in *Ci *that does not span *Ci*'s entire length. Since TR degeneracy can complicate identifying nested structures, XSTREAM only looks for nested TRs in consensus sequences. Our procedure detects nested TRs of unlimited nesting depth, with no gaps and no mismatches. This algorithm employs a top-down approach to locating TRs, as opposed to the bottom-up method used by XSTREAM. A top-down approach is useful for nested TRs because it identifies the longest period TR first, then in a recursive manner, restarts the algorithm within that TR, and continually digs deeper until no more TRs can be found. By working off the greedy assumption that the longest period TRs are the best candidates for nesting, we avoid issues of TR overlap inherent in the bottom-up strategy. The main drawback to our nesting method is its time complexity, which is O(*n*^3^), where *n *= *Pi*. We therefore restrict this method to TRs from {*X*} with periods ≤ 1000 and only find nested TRs with periods ≤ 300. We set the minimum nested TR period at 1 for proteins and 2 for nucleotide sequences. The time complexity is O(*n*^3^) due to the worst-case scenario of comparing subsequences of all possible sizes in all possible sequence regions.

### Divide and Conquer

XSTREAM implements a user-adjustable divide and conquer procedure to reduce memory consumption. If enabled, the input sequence is segmented into overlapping fragments of length *l *prior to TR detection. The last fragment of the input sequence may be of length <*l*. Overlapping regions have length l*, which is equivalent to the maximum detectable TR period. After all fragments are processed, the set of identified TRs are directed to the merging procedure, which functions to both extend TRs across fragment boundaries and consolidate overlapping regions. By default, *l *= 100,000 and l* = 10,000.
